# Advances in Single Cell Impedance Cytometry for Biomedical Applications

**DOI:** 10.3390/mi8030087

**Published:** 2017-03-12

**Authors:** Chayakorn Petchakup, King Ho Holden Li, Han Wei Hou

**Affiliations:** 1 Mechanical and Aerospace Engineering, Nanyang Technological University, Singapore 639798, Singapore; chayakor001@e.ntu.edu.sg; 2 Lee Kong Chian School of Medicine, Nanyang Technological University, Singapore 636921, Singapore; hwhou@ntu.edu.sg

**Keywords:** impedance spectroscopy, flow cytometry, single cell analysis

## Abstract

Microfluidics impedance cytometry is an emerging research tool for high throughput analysis of dielectric properties of cells and internal cellular components. This label-free method can be used in different biological assays including particle sizing and enumeration, cell phenotyping and disease diagnostics. Herein, we review recent developments in single cell impedance cytometer platforms, their biomedical and clinical applications, and discuss the future directions and challenges in this field.

## 1. Introduction

Single cell analysis has gained considerable attention for biological assays and system biology in the past decade due to the increasing importance of studying cell populations that are highly heterogeneous, as well as sampling of complex biofluids such as blood. Bulk measurement can only reflect the average value, leading to a loss of valuable information about rare sub-populations (diseased cells or abnormal cells) present in the sample [1,2]. In bio-related studies, coulter counter and fluorescence-activated cell sorting (FACS) are widely used as high throughput cell counting and classification methods. Coulter counter detects a change in direct current (DC) or low frequency alternating current (AC) impedance signal caused by particle or cell passing through the detection region which can provide information about particle size [3,4]. FACS is a more powerful technique and requires fluorescent cell labelling to enable counting, characterization and sorting based on optical characteristics. However, several drawbacks including laborious sample preparation, and expensive equipment and reagents (antibodies) significantly limit its use for point-of-care (POC) testing.

Characterization of electrical impedance at different frequencies provides important information about biological cells and the suspending medium, making it attractive tool for single cell analysis [5,6,7]. In the kHz range, there is α-dispersion which originates from the displacement of counterions around the charged shell of the cell. This is hard to measure as it is hindered by the effects of electrode polarization which leads to high impedance below 1 MHz [8]. β-dispersion occurring in MHz range arises from the interfacial polarization of cellular components such as the cell membrane. The polarization of protein and other organic molecules also contributes to different parts of β-dispersion [9]. In addition to cells and cellular components information, dynamic studies of red blood cell (RBC) aggregation [10] and blood coagulation [11] can also be obtained from this range. γ-dispersion above 1 GHz is due to dipolar relaxation of water bound molecules in the cytoplasm and the external medium [12]. 

Advances in microfluidics and biomedical microelectromechanical systems (BioMEMS) are important in the development of impedance cytometers as it enables manipulation of small fluid volumes, and highly-sensitive measurement with the close proximity of microfabricated electrodes to single cells in microchannels [13,14]. Besides reducing reagent and sample volume consumption and expensive equipment, a key advantage over the traditional methods is that sample preparation and impedance detection modules can be readily integrated in a single device, commonly known as lab-on-a-chip (LOC), for POC testing. 

In this review, we summarise recent developments of impedance based microfluidic cytometry for biomedical research. We will first provide a brief overview of the working principles and designs of impedance based microfluidic cytometry. For more detailed information, the readers can refer to other excellent reviews by Morgan et al. [15,16] and Chen et al. [17]. Next, we will focus on the diagnostics and phenotyping capabilities of impedance cytometry in different biomedical applications, and present reported work according to cell types including blood cells (leukocytes and RBCs), cancer cells, microbes and stem cells. Lastly, we will highlight future directions and challenges in this field based on our findings.

## 2. Design Principles

### 2.1. Theory 

Electrical impedance is defined as the ratio between excitation voltage and response current of cell in suspension,

(1) $$Z^{*}=\frac{V^{*}}{I^{*}}$$

where Z* is electrical impedance (Ohm), V* is excitation voltage (Volt) and I* is current response (Amp) and superscript * denotes complex number.

Various approaches have been utilized to simulate or to interpret impedance results of particle in suspension such as finite element method (FEM), Maxwell’s mixture theory (MMT) and equivalent circuit model (ECM). A comparison of three abovementioned approaches has been reported elsewhere [16,18,19,20,21,22].

Maxwell’s mixture theory describes the dielectric property of particle in suspension [23]. The complex permittivity of the mixture can be determined by three key parameters, which are the complex permittivity of the cell, complex permittivity of its suspending medium and volume fraction, which is the ratio of volume of the cell to the volume of the channel.

(2) $$\varepsilon_{\mathrm{mix}}^{*}=\varepsilon_{\mathrm{med}}^{*}\frac{2\left(1-\varphi \right)+\left(1+2\varphi \right)\frac{\varepsilon_{\mathrm{cell}}^{*}}{\varepsilon_{\mathrm{med}}^{*}}}{\left(2+\varphi \right)+\left(1-\varphi \right)\frac{\varepsilon_{\mathrm{cell}}^{*}}{\varepsilon_{\mathrm{med}}^{*}}}$$

where $\varepsilon^{*}=\;\varepsilon -j\frac{\sigma }{\omega }$ denotes complex permittivity, $j^{2}=-1,$ ω is the angular frequency, and φ is the volume fraction which is the ratio of volume of cell to volume of medium inside the detection channel. The subscript “mix”, “cell” and “med” represent mixture, cell and medium, respectively.

The complex permittivity of cell can also be determined in the same manner as complex permittivity of the mixture above. To determine complex permittivity of cell properly, several models have been proposed to describe cell or particle based on its internal complexity such as particle, single shelled model (cell consisting of cytoplasm and cell membrane) and double shelled model (cell consisting of cytoplasm, cell membrane and nucleus or vacuole) [24,25,26]. 

The impedance of the mixture containing cell (*Z*_mix_) can be calculated from the following equation.

(3) $$Z_{\mathrm{mix}}=\frac{1}{j{\omega \varepsilon }_{\mathrm{mix}}^{*}lG}$$

where *G* is a cell constant to correct the effect of non-uniform electric field and fringing field. *l* is width of the channel. The calculation of cell constant of different electrode configurations has been shown in previous literature [21,22,27,28,29]. 

### 2.2. Electrode Designs 

**Figure 1:**
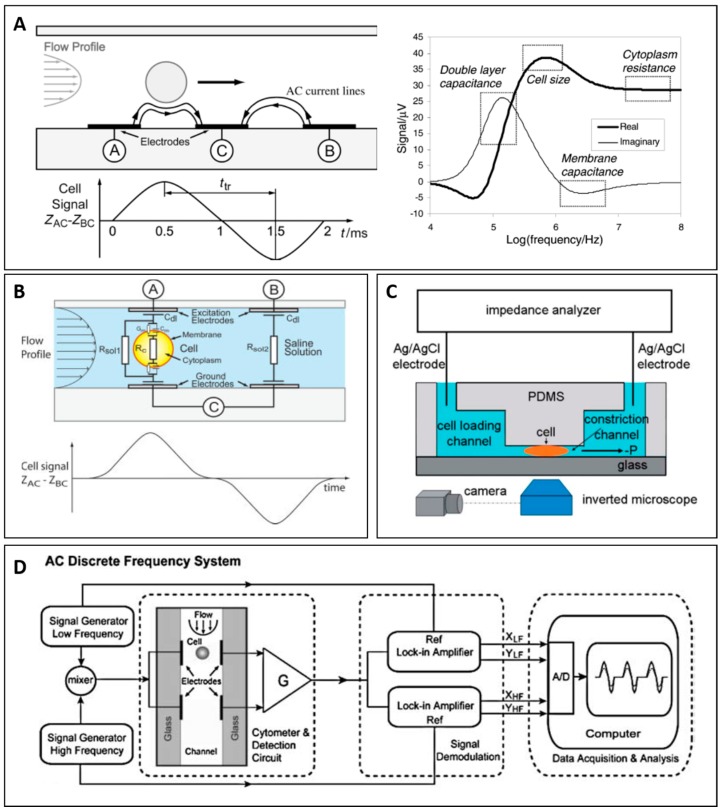
(**A**) (**Left**) Illustration of coplanar electrodes design and impedance signal response when a cell flows through the detection region. (**Right**) Impedance response at different frequencies carries different information regard the cell. Reproduced with permission from [30], copyright 2001, Royal Society of Chemistry. (**B**) Illustration of parallel electrodes design. Reproduced with permission from [31], copyright 2005, John Wiley and Sons. (**C**) Illustration of constriction channel design. Reproduced with permission from [32], copyright 2011, Royal Society of Chemistry. (**D**) Diagram shows the measurement setup. Reproduced with permission from [33], copyright 2008, Springer;

In this section, we describe three common configurations used in impedance based microfluidic cytometry: coplanar electrodes, parallel electrodes, and constriction channel. Figure 1A–C shows microfluidics impedance cytometers using coplanar electrodes design (Figure 1A), parallel electrodes design (Figure 1B), and constriction channel design (Figure 1C). Each design is based on a similar detection principle, with excitation electrode and sensing electrodes embedded inside microfluidic channel to establish electrical measurement. As a cell flows between a pair of electrodes (A and C), the electric field between these two electrodes is disrupted, resulting in a current change that can be measured at point A. The current measured at this position corresponds to the impedance of cell and its suspending medium. To determine impedance of medium, the current at point B is also acquired simultaneously and the impedance of cell can be acquired from the difference between current at point A and at point C (Figure 1A). Typically, the setup consists of pre-amplifier, lock-in amplifier and data acquisition system (Figure 1D). The excitation signal is supplied to excitation electrode by function generator or lock-in amplifier, and sensing electrodes are connected to bridge circuit or trans-impedance amplifiers to measure current response of system. The amplifiers’ output is connected to lock-in amplifier to demodulate current signal at excitation frequency. The data are sent to data acquisition system for post processing.

#### 2.2.1. Coplanar Electrode Design

Coplanar electrode configuration was first proposed by Gawad et al. [30]. In this design, coplanar metal electrodes were integrated in microchannel and non-homogeneous electric field was generated. The authors carried out the simulation of cell impedance from equivalent circuit model and their simulation result showed that different parts of impedance spectra contain different information of cell components as presented in Figure 1A. Furthermore, they showed that opacity or a ratio of high frequency impedance magnitude to low frequency impedance magnitude does not depend on position of cell in the channel. Since then, opacity is widely used as characterization parameter in impedance cytometry. 

The fabrication process of coplanar electrodes design starts with the patterning of electrode layer on glass substrate. The channel layer is then fabricated or bonded on glass substrate, creating a microfluidic device with integrated electrodes. The whole process can be easily fabricated since only a single alignment is needed to guide electrodes to the desirable position inside the channel.

**Figure 2:**
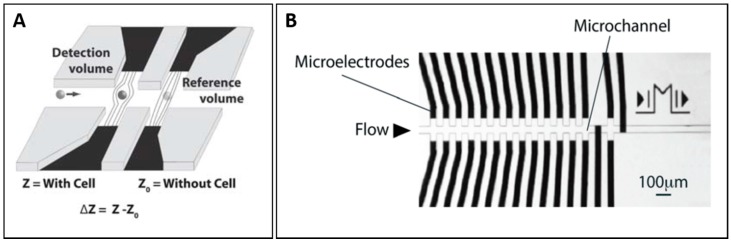
(**A**) Illustration of liquid electrode design. Reproduced with permission from [36], copyright 2010, Royal Society of Chemistry; and (**B**) illustration of combination approach of conventional coplanar electrode design and liquid electrode design. Reproduced with permission from [37], copyright 2014, Royal Society of Chemistry;

Due to non-uniform electric field created by coplanar electrode configuration, the impedance measurement relied on the vertical position of cell in the detection region considerably. To reduce the effect of vertical position of cell on impedance, another coplanar electrode configuration called liquid electrodes was used [34,35,36]. In this case, the electrodes were placed at bottom of lateral channels perpendicular to main channel, as shown in Figure 2A. As a result, homogeneous electrical field over the channel height was generated, mitigating the height dependence. However, this design had several drawbacks. Firstly, the sensitivity is poorer than traditional coplanar electrode design due to the increase in detection volume as the distance between the electrode pair needs to be placed far enough in order to generate homogenous electrical field across main channel. Secondly, the effect of lateral position rises due to fringing effect at edges of electrodes. In this work, they used dielectrophoresis (DEP) force generated by liquid electrodes to focus the cell at the centre of channel. Shaker et al. used the combination of conventional and liquid coplanar electrodes configuration shown in Figure 2B [37]. Longitudinal measurement and transverse measurement provide different characteristics that can be exploited to detect a shape of particle.

Besides down-scaling channel dimension to achieve higher sensitivity of coplanar electrodes, several techniques have been demonstrated to focus the cell to channel centre or control the vertical position of the cell in the channel using DEP [31,34,37,38,39,40] and hydrodynamic focusing [41,42,43,44,45,46]. 

For hydrodynamic focusing, there are two approaches: 1D hydrodynamic focusing [41,42,43] and 2D hydrodynamic focusing [44,45,46]. For these devices, the channels are typically larger and low conductivity sheath fluids such as deionized water [40,41,45] or oil [40,42] are used to achieve particle focusing. A three-inlet device was designed, in which two additional focalisation lateral inlets were used to provide focusing stream for pinching sample stream. Not only does it allow single particles to flow through detection region, but the detection volume between electrodes can also be adjustable to fit a wide range of particles or cells sizes. Moreover, the use of a large channel greatly reduces the chance of channel blockage. Besides utilization of 1D hydrodynamic focusing, 2D hydrodynamic focusing was adapted in several devices [44,45,46], aiming to control vertical position of cell leading to better sensitivity than 1D hydrodynamic focusing. 

To align particle to the centre of channel using DEP, several designs such as top and bottom taper shaped electrodes [39], coplanar deflecting electrodes [31] and liquid electrodes [34,37] have been reported. Noteworthy, the utilization of DEP focusing only provides the control of particle position in the channel, whereas hydrodynamic focusing can control both the particle position and detection volume.

Besides abovementioned particle focusing techniques, the effect of particle position on impedance can be also be corrected by multiple electrodes design and signal processing as demonstrated recently by De Ninno et al. [47]. Additional electrodes affect the measured signal profile which conveys information on particle position as well. Hence, the measured characteristic signal can be exploited to correct the signal of off-centre particle leading to accurate particle sizing. However, introduction of additional electrodes covers a larger region in the channel and a higher particle coincidence (two or more particles measured simultaneously) can occur if particle concentration is too high.

#### 2.2.2. Parallel Electrode Design

Parallel electrodes configuration was first developed by Gawad et al. [48]. In this configuration, electrodes were placed at top and bottom or at sidewall of microchannel. Similar to previous design, two pairs of electrodes were used to measure impedance of cell passing between electrodes and impedance of the medium as depicted in Figure 1B. With parallel electrodes, electric field distribution was less divergent, leading to better sensitivity as compared to coplanar electrode design. However, this design also suffers from the measured signal dependence on cell position inside detection volume [49].

The fabrication process is more complex as compared to coplanar electrodes design. For top and bottom electrodes configuration, two alignment steps are needed for aligning channel to electrode pattern and aligning two chips with electrodes together. Precise alignment is needed to make the measurement reproducible. For the sidewall electrodes configuration, sidewall electrodes were fabricated by electroplating followed by SU-8 channel fabrication on top of the electrodes. This can be done with single alignment. However, there is always a vertical gap between the sidewall electrodes and microchannel, resulting in an inhomogeneity of the electric field [50]. This can possibly lead to a slightly poorer performance as compared to top-bottom configuration.

Several studies utilized hydrodynamic focusing [42] and DEP to control particle position inside microchannel. Additionally, multi-electrodes design used to correct the signal of off-centre particles was proposed by Spencer et al. [51]. In this work, they used five pairs of parallel electrodes. However, unlike multiple coplanar electrodes design approach, this requires four transimpedance amplifiers to get parameters for correction, resulting in a more complicated setup.

#### 2.2.3. Constriction Channel Design

**Figure 3:**
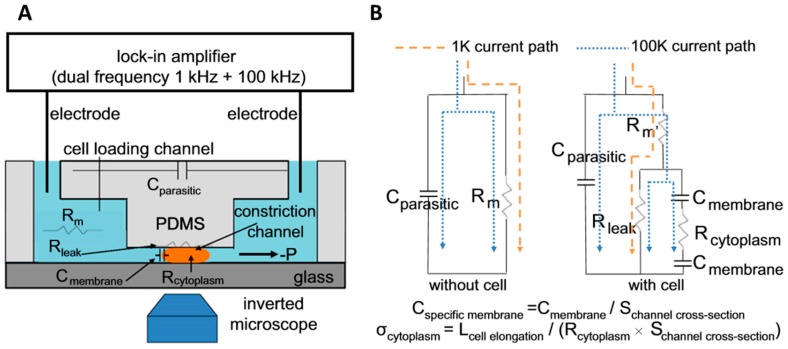
(**A**) Illustration of constriction channel design with dual frequencies measurement; and (**B**) equivalent circuit model and current paths at low and high frequency when constriction channel with cell and without cell. Reproduced with permission from [52], copyright 2013, Elsevier;

Lack of direct contact between electrodes and cell can introduce current leakage issues, in which current tends to pass though high conductivity fluid surrounding the cell. In order to solve this problem, the constriction channel design was introduced by Chen et al. [32]. In this design, the detection region was designed to be smaller than cell (Channel: 6 μm × 6 μm) as shown in Figure 3A. The Ag/AgCl electrodes placed at inlet and outlet were used instead of thin film electrodes on substrate. When cell was aspirated into the channel, the electric field across two electrodes was altered leading to the change in impedance which can be implied as impedance of cell. Moreover, mechanical properties such as cell deformability can be measured when the cell squeezes through the smaller channel, enabling multi-parametric mechanical and electrical cell characterization. Based on equivalent circuit model shown in Figure 3B, multi-frequencies measurement (at 1 kHz and 100 kHz) are used to determine size-independent electrical properties such as specific membrane capacitance (*C*_specific membrane_) and cytoplasm conductivity (σ_cytoplasm_). The drawbacks of this design are that it is prone to clogging and has lower throughput as compared to other designs.

The fabrication process of constriction channel design is simple, as only single alignment is needed in channel fabrication process. 

The comparison of each design is shown in Table 1.

## 3. Biomedical Applications

In this section, we highlight the biomedical applications of impedance cytometers based on cell types (Table 2).

### 3.1. Blood Cells

Previous works have used microfluidic impedance cytometry to study dielectric properties of various blood cells, including red blood cells [30,31,61,63,86] and white blood cells [18,38,44,50,53,55,56,57,58,59].

#### 3.1.1. White Blood Cells

**Figure 4:**
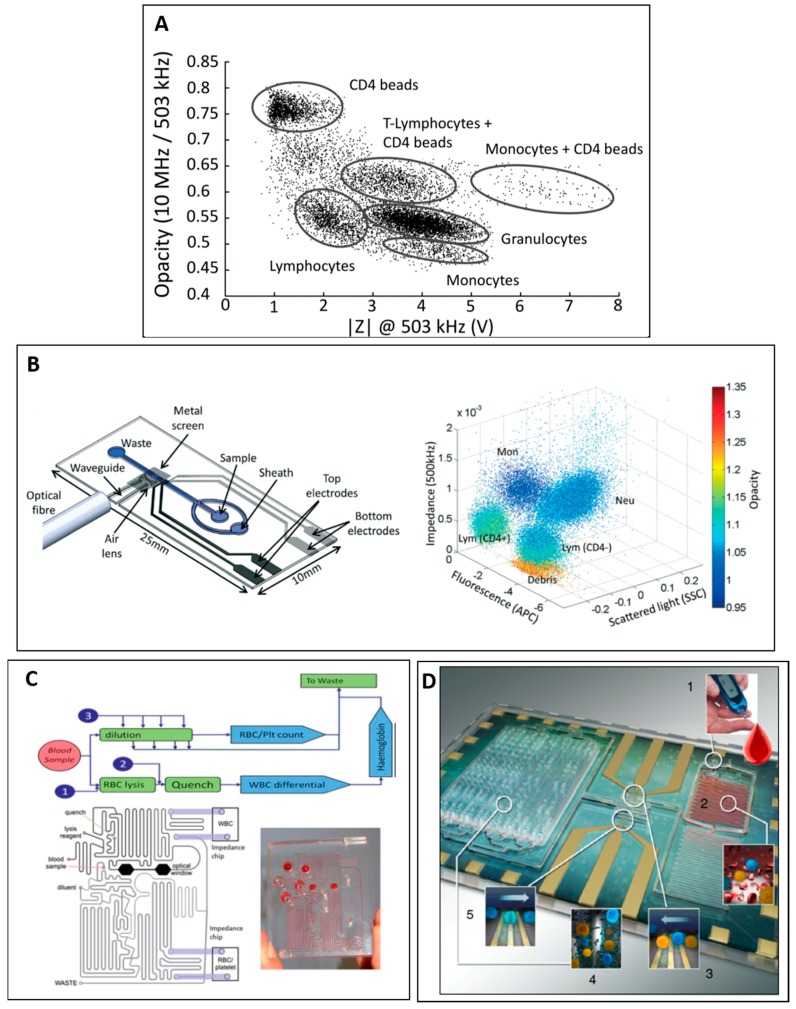
(**A**) Plot of opacity (10 MHz/503 kHz) versus impedance signal at 503 kHz for white blood cells population after addition of CD4 beads. Reproduced with permission from [53], copyright 2010, American Chemical Society. (**B**) (**Left**) Schematic of impedance cytometer with on-chip waveguide; and (**Right**) 3-D scatter plot of side scatter impedance and fluorescence for CD4 labelled white blood cells population. Colour represents opacity magnitude. Reproduced with permission from [58], copyright 2014, Royal Society of Chemistry. (**C**) Schematic of sample pretreatment module proposed by van Berkel et al. [55] for whole blood processing prior impedance detection. The design includes two pathways: (1) sample dilution followed by red blood cells and platelets counting; and (2) red blood cells lysis and quenching followed by white blood cells differential counting. Reproduced with permission from [55], copyright 2011, Royal Society of Chemistry. (**D**) Schematic of on-chip sample pretreatment with capture chamber for differential counting of CD4 or CD8: (**1**) inlets for loading of whole blood and reagent solutions (lysing buffer and quenching buffer); (**2**) lysing followed by quenching; (**3**) entrance counting; (**4**) CD4 or CD8 capture chamber; and (**5**) exit counter. Reproduced with permission from [59], copyright 2016, Nature Publishing Group;

Holmes et al. proposed impedance labelling technique for counting of CD4+ T-cells [53]. Anti-CD4 antibody coated beads (1.8–2.4 µm) were mixed with lysed whole blood and bound to the monocyte and CD4 expressing (CD4+) T-cells. As a result, the population of CD4+ cells were larger due to the beads bounded on their surfaces, resulting in an increase in opacity (10 MHz and 0.5 MHz) and impedance signal at 0.5 MHz (Figure 4A). This method of using impedance labelling enables enumeration of sub-population. Spencer et al. also described a novel sheathless microfluidic cytometer with on-chip waveguide (Figure 4B (left)) that can measure four parameters: fluorescence signal, large angle side scatter and impedance at two different frequencies (0.5 MHz and 2 MHz) (Figure 4B (right)).

In another application for whole blood enumeration, van Berkel et al. developed an integrated microfluidic platform with sample pretreatment module for a three-part differential leukocyte counting together with red blood cells and platelets counting [55]. Figure 4C shows sample pretreatment design. Blood sample supplied to the sample pretreatment module was divided into two branches: (1) dilution process of subsequent RBC and platelets counting based on impedance signal at 0.5 MHz; (2) RBC lysis and quenching followed by white blood cells (WBCs) discrimination based on opacity (1.7 MHz and 0.5 MHz) and impedance signal at 0.5 MHz. 

Recently, Hassan et al. reported a microfluidic impedance cytometer for simultaneous CD4+ and CD8+ T-cells counting [57,59,87]. In this design (Figure 4D), they included on-chip sample preparation and capture chamber specifically designed for capturing CD4+ or CD8+ T-cells. Cell population was electrically characterized before and after capture chamber, providing the number of cells captured in the capture chamber. The device was clinically validated in a cohort of healthy subjects and HIV+ patients, and they showed that the microfluidic measurements were strongly correlated to flow cytometry analysis. 

#### 3.1.2. Red Blood Cells and Platelets

Gawad et al. demonstrated the discrimination of normal red blood cells and ghost red blood cells (their cytoplasm replaced by hypotonic solution) based on impedance signal at 15 MHz, indicating the differences was due to cytoplasm conductivity between both RBCs populations [30].

**Figure 5:**
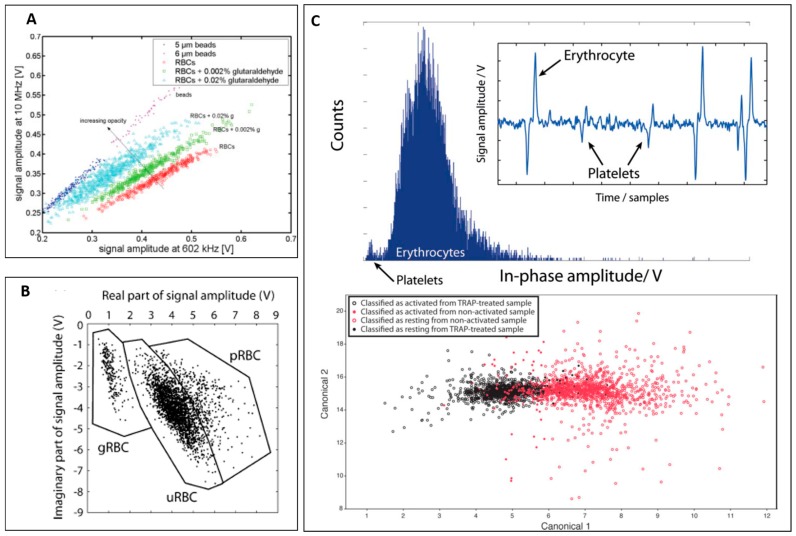
(**A**) Scatter plot of signal amplitude at 10 MHz versus 602 kHz for 5.6-µm beads, red blood cells and glutaraldehyde fixed red blood cells at different concentrations. Reproduced with permission from [31], copyright 2005, John Wiley and Sons. (**B**) Real part of signal amplitude versus imaginary part of signal magnitude of different kinds of red blood cells (ghost, normal, and parasite infected). Reproduced with permission from [61], copyright 2007, Elsevier. (**C**) (**Top**) Histogram shows the distribution of in-phase amplitude from platelets and red blood cells. (**Bottom**) Scatter plot of discriminant analysis of non-activated platelets and TRAP activated platelets. Reproduced with permission from [40], copyright 2013, Royal Society of Chemistry;

Cheung et al. used parallel electrodes design to distinguish three kinds of RBCs, healthy, ghost and glutaraldehyde-fixed, at different concentrations [31]. In this study, they showed the identification of RBCs and glutaraldehyde-fixed RBCs based on impedance signal at 10 MHz and 602 kHz (Figure 5A). The impedance signal at 10 MHz of fixed RBCs was higher than that of normal RBCs, indicating a decrease in cytoplasm conductivity or increase in opacity.

Parasite invasion of RBCs can alter dielectric properties of RBCs in malaria [61,63,88]. Kuttel et al. used coplanar electrodes design impedance cytometer to detect *Babesia bovis* infected red blood cells [61]. Figure 5B shows the difference in impedance signal at 8.7 MHz of normal red blood cells and *Babesia bovis* infected red blood cells.

For platelet analysis, Evander et al. demonstrated the detection of red blood cells and platelets as shown in Figure 5C (top) [40]. Moreover, the group also successfully classified non-activated platelets from thrombin receptor activating peptide (TRAP) activated platelets based on discriminant analysis from impedance signal at four frequencies (284 kHz, 1.20 MHz, 2.39 MHz and 4.02 MHz), which will be useful to study thrombosis or platelet dysfunctions (Figure 5C (bottom)).

### 3.2. Cancer Cells

Several impedance cytometers were developed to characterize various types of tumours and cancers [32,52,72,73,74,76,78,79].

**Figure 6:**
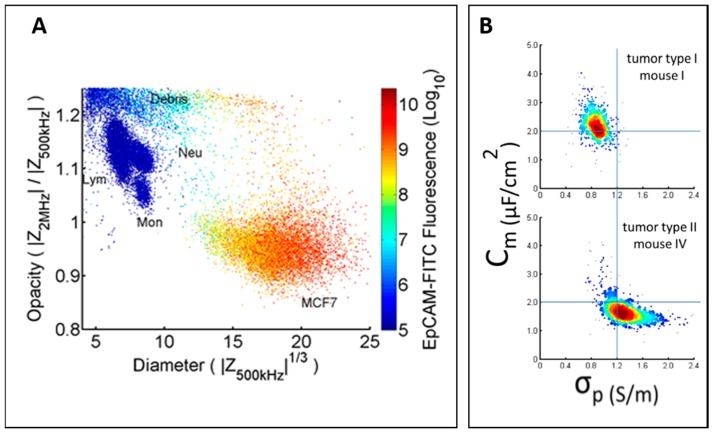
(**A**) Scatter plot of opacity (2 MHz/500 kHz) and diameter (impedance signal at 500 kHz) for leukocytes and MCF7 cells. Colour represents fluorescence signal. Reproduced with permission from [76], copyright 2014, AIP Publishing LLC. (**B**) Scatter plot of *C*_specific membrane_ versus σ_cytoplasm_ for A549 (mouse I) and H1299 (mouse IV). Reproduced with permission from [78], copyright 2016, Nature Publishing Group;

Previous studies reported distinct differences in dielectric properties of white blood cells and tumour cells, which generally have larger membrane capacitance and size [89,90]. Due to these differences, Spencer et al. demonstrated the use of parallel design microfluidic cytometer to distinguish breast tumour cells (MCF-7) from leukocytes when spiked in whole blood (Figure 6A) [76]. 

Zhao et al. characterized H1299 and A549 cells using a constriction channel design [78]. Specific membrane capacitance and cytoplasm conductivity of each population were acquired, enabling rapid discrimination of two tumour types (Figure 6B).

### 3.3. Microbes

Besides the discrimination of blood cells and tumours, impedance cytometry has also been utilized to characterize various kinds of samples such as yeast [37,66,68], bacteria [67] and plankton [64].

#### 3.3.1. Yeasts

**Figure 7:**
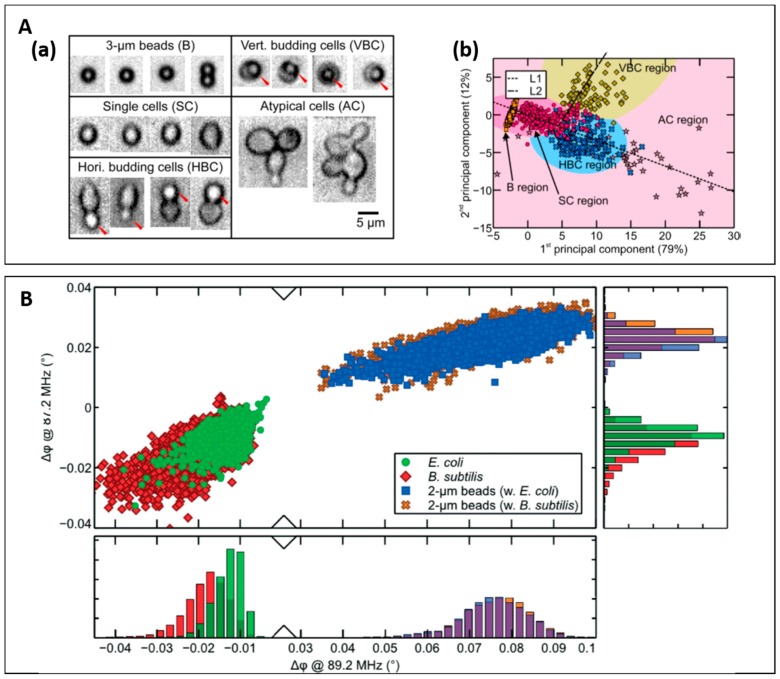
(**A**) (**a**) Different phenotypes of yeast cells used in Haandbæk’s studies [68]. (**b**) Scatter plot of the first two principal components as result from principal components analysis of the impedance information (impedance at four frequencies and velocity). Reproduced with permission from [68], copyright 2016, American Chemical Society. (**B**) The result from another Haandbæk’s studies. Scatter plot of phase shift at 87.2 MHz versus phase shift at 89.2 MHz for two experiments: (1) *E. coli* (green) spiked with 2-µm beads (blue); and (2) *B. subtilis* (red) spiked with 2-µm beads (yellow). Reproduced with permission from [67], copyright 2014, Royal Society of Chemistry;

Haandbæk et al. demonstrated the use of high frequency impedance (>50 MHz) to characterize wild-type yeast from a mutant based on impedance at 0.5 MHz and 100 MHz to reflect size and vacuole property [66]. They found that the distribution of mutant shifted toward a higher opacity magnitude (100 MHz to 0.5 MHz), indicating the difference in vacuole property or vacuole size. Interestingly, the difference in electrical volume profile at 0.5 MHz corresponded to the yeasts’ sub-populations (large mother cells and small daughter cells). In a follow-up study, they further investigated different yeast phenotypes based on impedance at four different frequencies (0.55–9.08 MHz), particle velocity and fluorescence signal (Figure 7A) [68].

#### 3.3.2. Bacteria

For bacteria detection, Haandbæk et al. reported a novel resonator enhanced impedance based cytometer for the detection of sub-micrometre beads and bacteria [67]. By adding a series resonator circuit at excitation part, the sensitivity at high frequency can be improved. Instead of using impedance magnitude, they used the phase shift (at 89.2 MHz) as the characterization parameters to distinguish bacteria and 2-µm beads. Interestingly, bacteria and 2-µm beads can be discriminated by using phase polarity at 87.2 MHz and 89.2 MHz due to difference in dielectric properties of their internal structure. However, the proposed technique cannot be used to distinguish different types of bacteria, as their dielectric properties of cytoplasm would be too similar (Figure 7B).

### 3.4. Stem Cells

Previous studies also showed the feasibility of using impedance cytometers to characterize stem cells differentiation [83,84,85].

**Figure 8:**
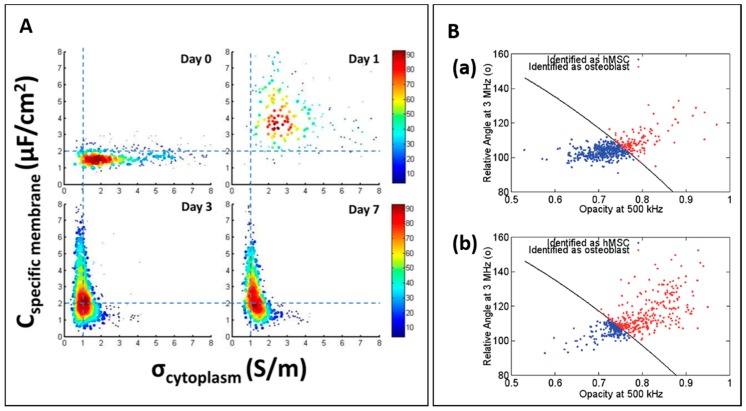
(**A**) Scatter plot of *C*_specific membrane_ versus σ_cytoplasm_ of cultured neural stem cells for different days. Reproduced with permission from [84], copyright 2016, Public Library of Science. (**B**) Classification results of human mesenchymal stem cells (hMSC) and osteoblast measured at: seven days (**a**); and 14 days (**b**). Reproduced with permission from [85], copyright 2016, Royal Society of Chemistry;

Zhao et al. studied the differentiation of neural stem cells using constriction channel based impedance cytometer [84]. In this study, murine neural stem cells were cultured and sampled for several days. Figure 8A shows the distribution of specific cell membrane capacitance and cytoplasm conductivity. Initially, the population had wide distribution of cytoplasm conductivity which corresponded to nature of collected neurospheres. Over time, the distribution of specific cell membrane capacitance changed continuously which indicates active changes in cell membrane of the population. These data suggest the potential of using electrical measurements to monitor cell differentiation process. 

Recently, Song et al. studied the differentiation states of mesenchymal stem cells [85]. In this study, human mesenchymal stem cells (hMSC) were induced to differentiate into osteoblasts and impedance was measured on Days 7 and 14 (Figure 8B). The classification model was trained by using relative angle at 3 MHz and opacity at 500 kHz of control hMSC and osteoblast population to determine osteoblast differentiation.

## 4. Conclusion and Future Directions

In this review, we present the developments of single cell impedance cytometry using microfluidics in the past decade. There are three developed designs: coplanar electrode design, parallel electrode design and constriction channel. Impedance cytometer can be utilized in a wide range of applications, from differential blood cell counting for disease diagnostics to monitoring cell phenotypic changes and microbial studies.

In terms of throughput, coplanar electrode design and parallel electrode design are much higher (~1000 cell/s) than constriction channel (~100 cells/s). Developing high-throughput cytometer to achieve traditional flow cytometry level remains a key challenge, as there are trade-offs between throughput and signal quality. Nevertheless, throughput can be increased using data acquisition with high sampling rate, or having multiple detection channels to further improve detection sensitivity and speed.

While most impedance measurements are based on cell size and membrane dielectric properties, impedance characterization of intracellular vacuole or nucleus is still at its infancy. Two possible reasons are the high frequency requirement (above 100 MHz) and difficulties in quantifying intracellular organelles position. In future studies, we envision that high frequency measurement of cells will be important as it can provide interesting insights about intracellular nucleus and organelles, which will be useful for developmental biology or genomics studies.

To facilitate user operations for biomedical applications, significant research efforts are focused on integrating important functionalities to microfluidic impedance cytometer such as optical detection, sample processing, and cell sorting. Optical detection such fluorescence labelling allows simultaneous characterization of cell phenotype with impedance measurement to study their associations, and further assess the potential of impedance-based biomarkers in clinical testing. Sample processing is another crucial feature for POC testing as most biofluids are complex and it is necessary to isolate the target cells prior analysis. Post measurement sorting is also attractive as it helps to further reduce the gap between microfluidic cytometer and conventional flow cytometry. Noteworthy, sorting based on impedance signature can be a label-free analytical tool which enables the separation of rare or abnormal cells without known good markers. 

In summary, there is a great potential of using impedance cytometer for biomedical applications and clinical diagnostics. In addition to technological improvements, large scale clinical validation will be necessary to determine feasibility of single cell impedance as novel biomarkers for disease diagnosis. 

## Figures and Tables

**Table 1:** Comparisons between different impedance cytometers design.

Design		Advantages	Disadvantages
Coplanar electrodes design	Coplanar electrodes	Simple fabrication	Vertical position dependence
		High throughput	Low sensitivity
	Liquid electrodes	Simple fabrication	Lateral position dependence
		High throughput	Low sensitivity ^1^
Parallel electrodes design	Top-Bottom configuration	High sensitivity	Vertical position dependence
		High throughput	Complex fabrication
	Sidewalls configuration	High sensitivity	Lateral position dependence
		High throughput	Complex fabrication
Constriction channel		Simple fabrication High sensitivity Size independent electrical parameters Mechanical property characterization	Prone to clogging Low throughput

^1^ Sensitivity comparison of each design was presented in several studies [30,34].

**Table 2:** List of developed impedance cytometry applications based on cell type. WBCs: white blood cells; RBCs: red blood cells; TRAP: thrombin receptor activating peptide; PDMS: polydimethylsiloxane.

Category	Author	Summary	Characterization parameters	Ref.
**White blood cells**	Watkins et al. (2009)	CD4 T-cells counting using impedance cytometer with 2D hydrodynamic focusing	Impedance at 50 kHz	[44]
	Holmes et al. (2009)	Discrimination of leukocyte subpopulation	Impedance signal at 1.7 MHz and 503 kHz	[18]
	Holmes et al. (2010)	Discrimination of T-cells and CD4 T-cells conjugated with CD4 beads	Impedance signal at 10 MHz and 503 kHz	[53]
	Watkins et al. (2011)	Differential count of CD4 T-cells by reverse-flow technique with integrated cell capture chamber	Impedance signal at 1.1 MHz	[54]
	van Berkel et al. (2011)	Differential counting of blood cells using impedance cytometer with off-chip sample treatment	Impedance signal at 1.7 MHz and 500 kHz	[55]
	Han et al. (2011)	Evaluation of RBC lysis chip for differential counting of WBCs	Impedance signal at 1.7 MHz and 444 kHz	[56]
	Watkins et al. (2013)	Integrated sample treatment and cell capture chamber for differential CD-4 and CD-8 T-cell counting	Impedance at 303 kHz and 1.7 MHz	[57]
	Spencer et al. (2014)	Integrated optical detection coupling with compound air lens for differential counting of blood cells	Impedance signal at 2 MHz and 500 kHz Fluorescence signal	[58]
	Frankowski et al. (2015)	Evaluation of parallel electrode designs for leukocyte sub-population counting	Impedance signal at 4 MHz and 500 kHz and fluorescence signal	[50]
	Hassan et al. (2016)	Integrated sample treatment and cell capture chamber for differential CD-4 and CD-8 T-cell counting	Impedance at 303 kHz and 1.7 MHz	[59]
**Red blood cells**	Gawad et al. (2001)	Discrimination of beads, RBCs and ghost RBCs	Impedance signal at 15 MHz and 1.72 MHz	[30]
	Cheung et al. (2005)	Discrimination of normal RBCs, ghost RBCs and glutaraldehyde fixed RBCs	Impedance signal at 10 MHz and 602 kHz	[31]
	Sun et al. (2007)	Utilization of maximum length sequences (MLS) for characterization of RBCs	Impedance spectrum up to 500 kHz	[60]
	Kuttel et al. (2007)	Discrimination of RBCs, B. Bovis infected RBCs and ghost RBCs	Impedance signal at 8.7 MHz	[61]
	Zheng et al. (2012)	Characterization of adult RBCs and neonatal RBCs	Transit time, amplitude ratio and phase shift at 100 kHz	[62]
	Du et al. (2013)	Discrimination of different states of Plasmodium falciparum infected RBCs	Combination of phase shift and magnitude shift in impedance at 2 MHz	[63]
	Evander et al. (2013)	Discrimination of RBCs, platelets and TRAP treated platelets.	Impedance signal at 284 kHz, 1.20 MHz, 2.39 MHz and 4.02 MHz	[40]
**Microbes**	Benazzi et al. (2007)	Discrimination of three different types of phytoplankton	Impedance signal at 6 MHz and 327 kHz and fluorescence signal	[64]
	Rodriguez-Trujillo et al. (2007)	Discrimination of 20 μm polystyrene beads and 5 μm yeast cells	Impedance at 120 kHz	[45]
	Bernabini et al. (2010)	Discrimination of 1 μm, 2 μm beads and *Escherichia coli*	Impedance signal at 503 kHz	[42]
	Mernier et al. (2012)	Characterization of yeast cells before and after electrical lysis or thermal lysis	Impedance signal at 10 kHz	[65]
	Shaker et al. (2014)	Single cell morphology discrimination of budding yeasts’ division stage by using liquid electrodes	Impedance signal at 427 kHz and 533 kHz	[37]
	Haandbaek et al. (2014)	Discrimination of wild-type yeasts and mutant yeasts	Impedance signal at 100 MHz and 0.5 MHz	[66]
	Haandbaek et al. (2014)	Discrimination of bacteria (*E. coli* and *B. subtilis*) and 2 μm beads by using resonator circuit	Signal polarity at 87.2 MHz and 89.2 MHz	[67]
	Haandbaek et al. (2016)	Discrimination of single and budding yeast cells	Impedance signal at 20 MHz, 9 MHz, 1 MHz and 0.55 MHz	[68]
**Tumors**	Schade-Kampmann et al. (2008)	Discrimination of mouse fibroblast, adipocytes, human monocytes, dendritic cells and macrophages	Impedance signal at 2 MHz, 5 MHz and 14 MHz	[69]
	Nikolic-Jaric et al. (2009)	Discrimination of different sized polystyrene beads, yeast cells and Chinese hamster ovary (CHO) cells	Capacitance at 1.5 GHz	[70]
	Gou et al. (2011)	Discrimination of liver tumour cells at normal, apoptotic and necrotic status and leukaemia cells	Resistance and capacitance change at 100 kHz	[71]
	Chen et al. (2011)	Characterization of osteoblasts and osteocytes/EMT6 cells and EMT6/AR1.0 cells	Cell elongation, transit time and impedance amplitude ratio at 100 kHz	[32]
	Mernier et al. (2012)	Utilization of lateral liquid electrodes for focusing and for discrimination of live and dead CHO cells	Impedance signal at 500 kHz and 15 MHz	[34]
	Zheng et al. (2012)	Characterization of 3249 AML-2 cells and 3398 HL-60 cells	Membrane capacitance and cytoplasm conductivity	[72]
	Zhao et al. (2013)	Characterization of kidney tumour cells (786-O) and vascular smooth muscle cells (T2)	Membrane capacitance and cytoplasm conductivity	[52]
	Zhao et al. (2013)	Characterization of lung cancer cell lines (CRL-5803 cells and CCL-185)	Membrane capacitance and cytoplasm conductivity	[73]
	Zhao et al. (2014)	Characterization of various kinds of tumour such as 95C and 95D/549 and A549 CypA-KD	Membrane capacitance and cytoplasm conductivity	[74]
	Kirkegaard et al. (2014)	Characterization of HeLa cells and Paclitaxel treated HeLa cells	Impedance signal at 1.57 MHz and 82 kHz	[75]
	Spencer et al. (2014)	Detection of MCF7 cells spiked in whole blood	Impedance signal at 4 MHz and 500 kHz	[76]
	Bürgel et al. (2015)	Inversion of flow direction enabling impedance measurement of HeLa and CHO-K1 cells before and after electroporation	Impedance spectra from 20 kHz to 20 MHz with 8 steps	[77]
	Zhao et al. (2015)	Characterization of mouse tumour cell lines (A549 and H1299)	Membrane capacitance and cytoplasm conductivity	[78]
	Huang et al. (2015)	Characterization of normal PC-3 cells and PC-3 cells with membrane staining and/or fixation (4 conditions)	Membrane capacitance and cytoplasm conductivity	[79]
	Yuan et al. (2016)	Utilization of Ag PDMS as sidewall electrodes for discrimination of AML-2 and HL-60	Impedance signal ranging from 11 kHz–6 MHz	[80]
	Babahosseini et al. (2016)	Study the effect of different drug delivery approaches on electrical properties of MDA-MB-231	Impedance at 1 kHz, 10 kHz, 100 kHz and 1 MHz	[81]
	Xie et al. (2017)	Discrimination of apoptotic, necrotic and live HeLa cells	Conductance and susceptance at 1 MHz	[82]
**Stem cells**	Song et al. (2013)	Characterization of mouse embryonic carcinoma cell (P19) differentiation	Impedance signal at 50 kHz, 250 kHz, 500 kHz and 1 MHz	[83]
	Zhao et al. (2016)	Characterization of neural stem cell in differentiation	Membrane capacitance and cytoplasm conductivity	[84]
	Song et al. (2016)	Characterization of human mesenchymal stem cells and osteoblasts	Opacity at 500 kHz and relative angle at 3 MHz	[85]
